# A platform for glycoengineering a polyvalent pneumococcal bioconjugate vaccine using *E. coli* as a host

**DOI:** 10.1038/s41467-019-08869-9

**Published:** 2019-02-21

**Authors:** Christian M. Harding, Mohamed A. Nasr, Nichollas E. Scott, Guillaume Goyette-Desjardins, Harald Nothaft, Anne E. Mayer, Sthefany M. Chavez, Jeremy P. Huynh, Rachel L. Kinsella, Christine M. Szymanski, Christina L. Stallings, Mariela Segura, Mario F. Feldman

**Affiliations:** 1 VaxNewMo LLC, St. Louis, MO 63108 USA; 2 Department of Biological Sciences, University of Alberta, Edmonton, AB T6G 2R3 Canada; 3 Department of Microbiology and Immunology, Institute for Infection and Immunity, University of Melbourne at the Peter Doherty, Parkville, VIC 3010 Australia; 4 Swine and Poultry Infectious Diseases Research Center, Faculty of Veterinary Medicine, University of Montreal, 3200 Sicotte Street, St-Hyacinthe, QC J2S 2M2 Canada; 5 Department of Molecular Microbiology, Washington University School of Medicine, St Louis, MO 63110 USA; 6 Department of Microbiology and Complex Carbohydrate Research Center, University of Georgia, Athens, GA 30602 USA; 7 Present Address: Department of Biology, Centre for Applied Synthetic Biology, Concordia University, Montreal, QC H4B 1R6 Canada

## Abstract

Chemical synthesis of conjugate vaccines, consisting of a polysaccharide linked to a protein, can be technically challenging, and in vivo bacterial conjugations (bioconjugations) have emerged as manufacturing alternatives. Bioconjugation relies upon an oligosaccharyltransferase to attach polysaccharides to proteins, but currently employed enzymes are not suitable for the generation of conjugate vaccines when the polysaccharides contain glucose at the reducing end, which is the case for ~75% of *Streptococcus pneumoniae* capsules. Here, we use an *O*-linking oligosaccharyltransferase to generate a polyvalent pneumococcal bioconjugate vaccine with polysaccharides containing glucose at their reducing end. In addition, we show that different vaccine carrier proteins can be glycosylated using this system. Pneumococcal bioconjugates are immunogenic, protective and rapidly produced within *E. coli* using recombinant techniques. These proof-of-principle experiments establish a platform to overcome limitations of other conjugating enzymes enabling the development of bioconjugate vaccines for many important human and animal pathogens.

## Abstract (web summary)

Bioconjugation is a promising process to manufacture conjugate vaccines, but currently employed enzymes cannot generate the full spectrum of bacterial glycoproteins. Here, the authors use an O-linking oligosaccharyltransferase to generate a polyvalent pneumococcal bioconjugate vaccine with polysaccharides containing glucose at their reducing end.

## Introduction

S*treptococcus pneumoniae* (pneumococcus) is a leading cause of bacterial-induced pneumonia, meningitis, and bacteremia globally, particularly, afflicting children 5 years of age or younger^1,2^. Moreover, a 2000 epidemiological survey from the World Health Organization (WHO) estimated that 735,000 human immunodeficiency virus-uninfected children died from pneumococcal-related diseases^2^ with updated estimates slightly reduced to 541,000 deaths for the year 2008 (ref. ^3^). An increase in the number of prophylactic treatment options, mainly due to advancements in pneumococcal vaccine developments, has emerged over the past two decades. Pneumovax^®^23, a 23-valent polysaccharide vaccine, is used in elderly populations as well as children over the age of 2 years who are at increased risk of pneumococcal disease;^4^ however, polysaccharide vaccines typically act as T cell-independent antigens and are generally not effective in children 2 years of age and younger^5^. On the other hand, covalently linking a polysaccharide to a protein in the form of a conjugate vaccine elicits a T cell-dependent immune response across all age groups, characterized by high-affinity immunoglobulin G (IgG)-producing plasma cells and memory B cells^6,7^.

Three pneumococcal conjugate vaccines have been commercially licensed since the year 2000: Prevnar^®^, Synflorix™, and Prevnar 13^®^. Prevnar 13^®^, the most broadly protecting pneumococcal conjugate vaccine, is comprised of 13 protein-polysaccharide conjugates consisting of pneumococcal serotypes 1, 3, 4, 5, 6A, 6B, 7F, 9V, 14, 18C, 19A, 19F, and 23F, each individually linked to the genetically inactivated diphtheria toxoid CRM_197_. Although highly protective in a three-dose schedule, Prevnar 13^®^ is one of the most expensive vaccines on the market today. This is mainly due to its complex manufacturing process resulting in a cost of ~600 US dollars for primary and booster immunizations^8^. In fact, Prevnar 13^®^ has been Pfizer’s best-selling product for the fiscal years 2015–2017, with total revenues exceeding 17.5 billion US dollars^9^. Although pneumococcal conjugate vaccines have significantly reduced the burden of pneumococcal disease events^10,11^, due to variations in global serotype distributions^12,13^, serotype replacement events^14^, as well as the lack of a low-cost alternative for developing countries, alternative manufacturing strategies to expedite development of next generation vaccines are needed.

As mentioned above, currently licensed pneumococcal conjugate vaccines are synthesized chemically, which is a laborious process plagued with technical challenges, low yields, and batch-to-batch variations^15^, highlighting the need for improved conjugate vaccine synthetic methodologies. Over the past 15 years, in vivo conjugation using bacterial protein glycosylation systems has emerged as a feasible alternative to chemical conjugation^16^, with multiple bioconjugate vaccine candidates now in various stages of development and clinical trials^17,18^. Bioconjugation is based on exploiting protein glycosylation, a ubiquitous post-translational modification in which glycans are covalently linked to proteins. In bacteria, glycans are commonly bound to proteins via *N*- or *O*-linkages on asparagine or serine/threonine residues, respectively^19,20^. Several pathways for bacterial glycosylation have been characterized, and among the best described are the oligosaccharyltransferase (OTase)-dependent pathways in Gram-negative bacteria^20^. In these systems, a lipid-linked oligosaccharide is assembled sequentially at the cytoplasmic leaflet of the inner membrane, flipped to the periplasmic leaflet, and then transferred to acceptor proteins by either *N*- or *O*-OTases depending on the site of glycan attachment^20^. Many bacterial species, including *S. pneumoniae*, also synthesize capsular polysaccharides (CPSs) employing the same lipid-linked oligosaccharides prior to their polymerization, export, and transfer to the cell surface enabling their exploitation for bioconjugation reactions in *Escherichia coli*^21^.

Glycoproteins have been recombinantly synthesized in *E. coli* for use as vaccines^16^ and/or diagnostics^22,23^ by co-expressing three components: a genetic cluster encoding for the proteins required to synthesize a glycan of interest, an OTase and an acceptor protein. One drawback of this process is the apparent glycan substrate specificity of the known OTases, which, for some of them, has been suggested to be dictated by the reducing end sugar^24^ (the first monosaccharide in the growing polysaccharide chain) of the lipid-linked oligo/polysaccharide of interest. Although OTases are able to transfer many different oligo- and polysaccharide structures^24,25^, some sugars have not been efficiently conjugated by known OTases to acceptor proteins. Therefore, characterizing different OTases is paramount for expanding our arsenal of therapeutic glycoproteins, including bioconjugate vaccines.

OTases currently used for commercially synthesizing glycoconjugates are the *Campylobacter jejuni N*-OTase PglB^16^ and the *Neisseria meningitidis O*-OTase PglL^26^, both of which exhibit a great deal of promiscuity towards glycan substrates^24,25^. However, neither enzyme has been experimentally demonstrated to conjugate glycans containing a glucose residue at the reducing end, such as ~75% of *S. pneumoniae* CPSs^19,27^. In the present work, we demonstrate the first successful in vivo conjugation of *S. pneumoniae* CPSs containing glucose as the reducing end monosaccharide from multiple serotypes. This has been achieved using a different class of *O*-OTase, previously designated as PglL_ComP_ by our group^28^, and henceforth termed PglS. Here, we present proof-of-concept studies on the engineering, characterization, and immunological responses of a polyvalent pneumococcal bioconjugate vaccine using the natural acceptor protein ComP as a vaccine carrier as well as a monovalent pneumococcal bioconjugate vaccine using a conventional vaccine carrier containing the *Pseudomonas aeruginosa* exotoxin A protein.

## Results

### PglS transfers pneumococcal CPS14 to its acceptor protein

**Fig. 1:**
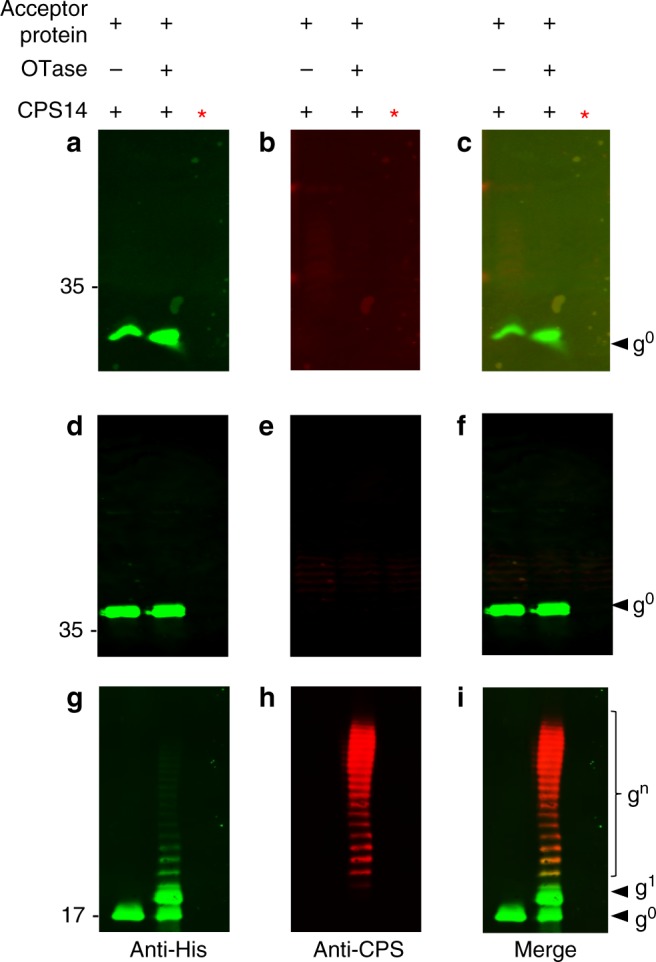
PglS can glycosylate the acceptor protein ComP with the pneumococcal CPS14 polysaccharide. *Escherichia coli* SDB1 cells co-expressing an acceptor protein (DsbA, AcrA, or ComP), an OTase (PglL, PglB, or PglS), and the CPS14 polysaccharide were analyzed for protein glycosylation via western blot analysis of the affinity-purified acceptor proteins. **a**–**c** DsbA purified from SDB1 cells in the presence or absence of PglL. **a** Anti-His channel probing for Hexa-histidine tagged DsbA. **b** Anti-glycan channel probing for CPS14. **c** Merged images for panels a and b. **d**–**f** AcrA purified from SDB1 cells in the presence or absence of PglB. **d** Anti-His channel probing for Hexa-histidine tagged AcrA. **e** Anti-glycan channel probing for CPS14. **f** Merged images for panels d and e. **g**–**i** ComP purified from SDB1 cells in the presence or absence of PglS. **g** Anti-His channel probing for Hexa-histidine-tagged ComP. **h** Anti-glycan channel probing for CPS14. **i** Merged images for panels g and h. The red asterisk indicates samples that were proteinase K treated for 1 h at 55 °C. Source data are provided as a Source Data file

PglB, the first OTase described, was shown to preferentially transfer glycans containing an acetamido group at the C-2 position of the reducing end sugar to asparagine residues of acceptor proteins^24^. However, polysaccharides with galactose (Gal) at the reducing end, such as the *Salmonella enterica* Typhimurium O antigen, have been transferred by an engineered PglB variant^29^ and also by PglL, the *O*-OTase from *Neisseria meningiditis*^25^. However, there is no evidence available for PglB- or PglL-mediated transfer of polysaccharides containing glucose (Glc) at the reducing end. We therefore tested the ability of PglB and PglL to transfer the pneumococcal CPS14, which has a Glc residue as the reducing end sugar, to their cognate acceptor proteins, AcrA and DsbA, respectively. As seen in Fig. 1a–f, no evidence for CPS14 glycosylation to either acceptor protein was observed.

Previously, we demonstrated that *Acinetobacter* species contain three *O-*linked OTases: a general PglL OTase responsible for glycosylating multiple proteins, and two pilin-specific OTases^28^. The first pilin-specific OTase is an ortholog of TfpO (also known as PilO) and is not employed for in vivo conjugation systems due to its inability to transfer polysaccharides with more than one repeating unit^26^. The second pilin-specific OTase, PglS, glycosylates a single protein, the type IV pilin ComP^28^. A bioinformatic analysis indicated that PglS is the archetype of a distinct family of OTases, which prompted us to test its ability to transfer pneumococcal CPS14 to ComP. Western blotting analysis (Fig. 1g–i) showed that co-expression of the CPS14 biosynthetic locus in conjunction with PglS and a His-tagged variant of ComP resulted in a typical ladder-like pattern of bands compatible with protein glycosylation with multiple subunits. Both ComP-His (Fig. 1g) and CPS14 (Fig. 1h) were detected with antisera specific to each antigen; moreover, samples treated with proteinase K did not react with either the anti-His or anti-CPS14 antisera, indicating that the purified material is indeed proteinaceous. Together, these results suggest that, unlike the previously characterized OTases, PglS is able to transfer polysaccharides with Glc at the reducing end.

### ComP is glycosylated at a serine residue in position 84

**Fig. 2:**
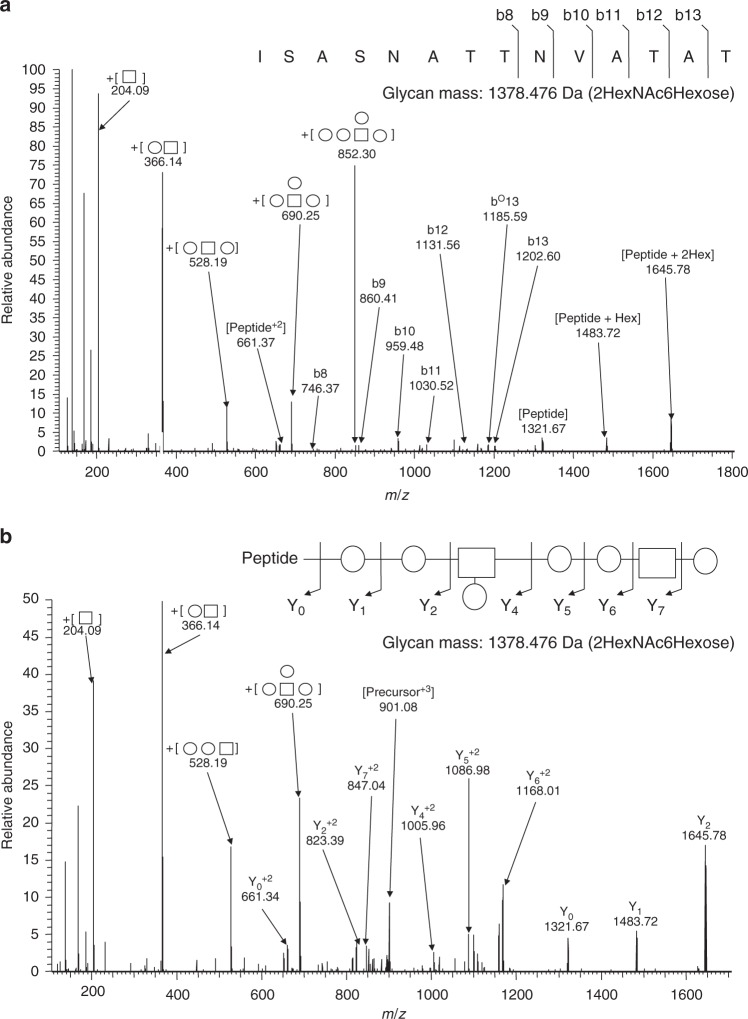
Higher energy collisional dissociation (HCD) fragmentation spectra of GluC-digested CPS14-ComP bioconjugates. GluC-digested CPS14-ComP was subjected to HCD fragmentation enabling the confirmation of a single peptide attached to a glycan with the CPS14 repeating subunit. High collision energies (**a**) and low collision energies (**b**) regimens were undertaken to confirm the glycosylation of the peptide _81_ISASNATTNVATAT_94_ with a 1378.47 Da glycan corresponding to HexNAc_2_Hexose_6_

*N*-glycosylation in bacteria generally occurs within the sequon D-X-N-S-T, where X is any amino acid but proline^30^. On the contrary, *O*-linked OTases do not seem to have defined recognition sequons. Most *O*-glycosylation events in bacterial proteins occur in regions of low complexity (LCR), rich in serine, alanine, and proline residues^31,32^. Some pilins are also *O*-glycosylated at a C-terminal serine residue^33^. We were unable to find an obvious LCR or a C-terminal serine residue in ComP homologous to those found in other pilin-like proteins and therefore employed mass spectrometry to determine the site(s) of glycosylation. Purified CPS14-ComP bioconjugates were subjected to GluC proteolytic digestion and multiple mass spectrometric analyses. As seen in Fig. 2, we identified a single glycopeptide consisting of a semi-GluC-derived peptide _81_ISASNATTNVATAT_94_ attached to a glycan that matched the published CPS14 composition (Fig. 2a). To enable the confirmation of both the peptide and attached glycan sequences, multiple collision energies regimens were performed to confirm the glycosylation of the semi-GluC-derived peptide _81_ISASNATTNVATAT_94_ with a 1378.47 Da glycan corresponding to HexNAc_2_Hexose_6_ (Fig. 2b). Additional glycopeptides were also observed decorated with extended glycans corresponding to up to four tetrasaccharide repeat units (Supplemental Fig. 1).

We have previously shown that *Acinetobacter* species predominantly glycosylate proteins at serine residues and thus hypothesized that either serine 82 or 84 was the site of glycosylation^32^. To determine which serine residue was the site of glycan attachment, we employed the *C. jejuni* heptasaccharide as the donor glycan, due to the ease at which glycosylation is detectable from whole-cell lysates. Wild-type ComP was glycosylated with the *C. jejuni* heptasaccharide as indicated by its increased electrophoretic mobility and signal co-localization with hR6 anti-glycan sera when co-expressed with PglS (Supplemental Fig. 2a-c). Mass spectrometry (MS) analysis also confirmed the presence of the *C. jejuni* heptasaccharide on the identical semi-GluC-derived peptide _81_ISASNATTNVATAT_94_ modified by CPS14 (Supplemental Figs. 3 and 4). As a negative control, we generated a catalytically inactive PglS mutant (H324A), which when co-expressed with the *C. jejuni* heptasaccharide glycan was unable to glycosylate wild-type ComP (Supplemental Fig. 2A-C). We next performed site-directed mutagenesis and observed that glycosylation of ComP with the *C. jejuni* heptasaccharide was abolished in the ComP[S84A] mutant, whereas ComP[S82A] was glycosylated at wild-type levels (Supplemental Fig. 2A-C). In addition, the site of ComP glycosylation was also determined using a pneumococcal polysaccharide and is discussed below.

### Immunogenicity of a monovalent CPS14-ComP bioconjugate

We evaluated the immunogenicity of a CPS14-ComP bioconjugate in a murine vaccination model. Two groups of mice (*n* = 10) individually received 3 µg of either unglycosylated ComP or CPS14-ComP bioconjugate. Mice were boosted on days 14 and 28, and sacrificed on day 49 for whole-blood collection. Each vaccine was formulated based on total protein. Using an enzyme-linked immunosorbent assay (ELISA) with a serotype 14 strain of *S. pneumoniae* adsorbed to each well, we compared IgM and IgG responses to CPS14. As seen in Supplemental Fig. 5, sera collected from mice vaccinated with a CPS14-ComP bioconjugate had an increased IgG response specific to CPS14 (Supp. Figure 5B), but not an increased IgM response (Supp. Figure 5A). Further, we employed secondary horseradish peroxidase (HRP)-tagged anti-IgG subtype antibodies to determine which of the IgG subtypes were present in CPS14-ComP-vaccinated mice (Supp. Figure 5C). We determined that the CPS14-specific IgG1 response was higher than the other subtypes, which is consistent with previous findings for pneumococcal conjugate vaccines^34,35^.

### Immunogenicity of a trivalent pneumococcal bioconjugate

**Fig. 3:**
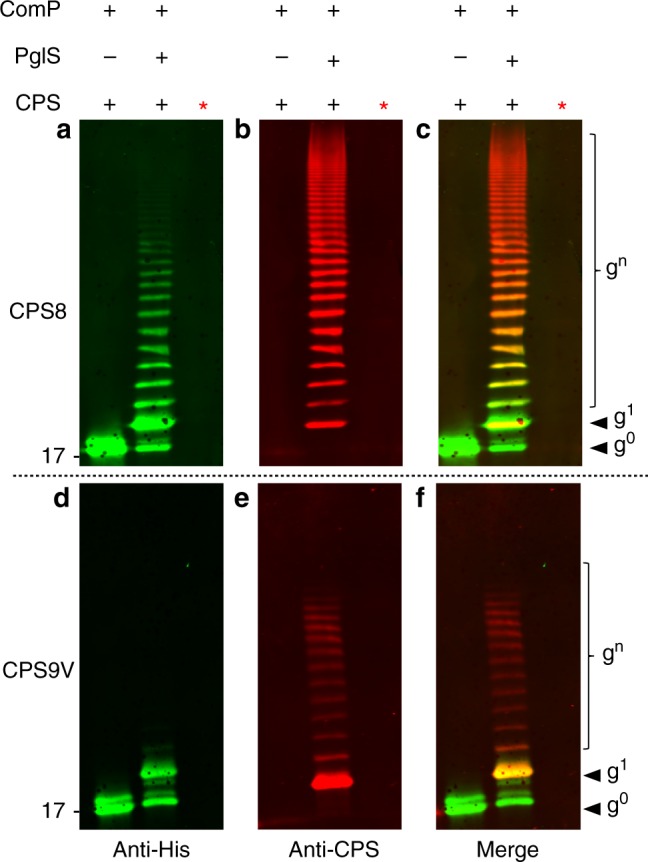
Western blot analysis of CPS8-ComP and CPS9V-ComP glycoproteins. *Escherichia coli* SDB1 cells were prepared co-expressing ComP, PglS, and either the pneumococcal CSP8 or CPS9V. Affinity-purified glycosylated ComP from each strain was analyzed for protein glycosylation via western blot analysis. **a***–***c** Western blot analysis of CPS8-ComP bioconjugates compared against ComP alone. **a** Anti-His channel probing for Hexa-histidine-tagged ComP purified from SDB1 expressing CPS8 in the presence or absence of PglS. **b** Anti-glycan channel probing for CPS8. **c** Merged images for panels a and b. **d***–***f** Western blot analysis of CPS9V-ComP bioconjugates compared against ComP alone. **d** Anti-His channel probing for Hexa-histidine-tagged ComP purified from SDB1 expressing CPS9V in the presence or absence of PglS. **e** Anti-glycan channel probing for CPS9V. **f** Merged images for panels d and e. The red asterisk indicates samples that were proteinase K treated for 1 h at 55 °C. Source data are provided as a Source Data file

There are more than 90 serotypes of *S. pneumoniae*^21,27^. Many increasingly prevalent serotypes, like serotypes 8, 22F, and 33F, are not included in currently licensed vaccines^36^. Therefore, we tested the versatility of PglS to generate a multivalent pneumococcal bioconjugate vaccine against two serotypes included in Prevnar 13^®^ (serotypes 9V and 14) and one serotype not included (serotype 8). The aforementioned CPSs all contain Glc as the reducing end sugar and are therefore not compatible with other commercially exploited conjugating enzymes. As seen in Figs. 3a-c and 3d-f, western blot analyses of affinity-purified proteins from whole cells co-expressing PglS, ComP, and either the CPS8 or CPS9V polysaccharides resulted in the generation CPS-specific ComP bioconjugates, respectively. Again, to confirm that the material purified was not contaminated with lipid-liked polysaccharides, we treated the samples with proteinase K and observed a loss of signal when analyzed via western blotting, confirming that the bioconjugates were proteinaceous.

**Fig. 4:**
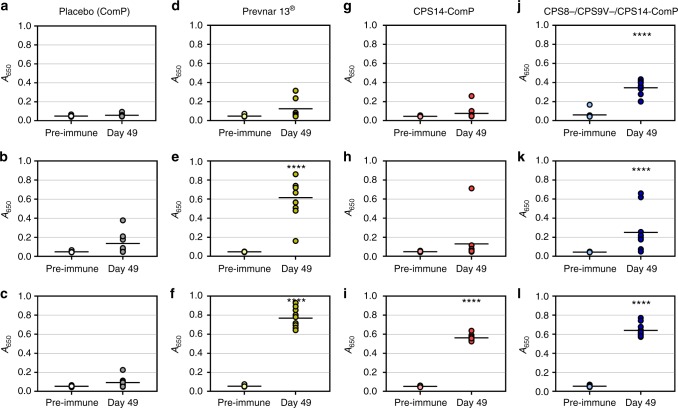
Immunoglobulin G (IgG) responses of mice vaccinated with ComP, Prevnar 13^®^, a monovalent bioconjugate and a trivalent bioconjugate. Groups of mice were vaccinated with ComP alone, Prevnar 13^®^, a monovalent CPS14-ComP bioconjugate vaccine, or a CPS8-/CPS9V-/CPS14-ComP biconjugate vaccine. Sera were collected on day 49 and analyzed for serotype-specific IgG responses via enzyme-linked immunosorbent assay (ELISA) compared against sera collected on day 0. **a**–**c** No IgG responses were detected in placebo vaccinated-mice for serotypes 8 (**a**), 9V (**b**), or 14 (**c**). **d**–**f** Prevnar 13^®-^vaccinated mice did not have detectable IgG responses to serotype 8 (**d**), but did have IgG responses specific to serotype 9V (**e**) and 14 (**f**). **g**–**i** Mice vaccinated with a CPS14-ComP bioconjugate vaccine did not have IgG responses to serotypes 8 (**g**) or 9V (**h**), but did have IgG responses to serotype 14 (**i**). **j**–**l** Trivalent CPS8-/CPS9V-/CPS14-ComP bioconjugate vaccinated mice all had statistically significant IgG responses to serotypes 8 (**j**), 9V (**k**), and 14 (**l**). Unpaired *t* tests (Mann–Whitney) were performed to statistically analyze pre-immune sera from day 49 sera. *P* values for each case tested were ****p = 0.0001. Each dot represents a single vaccinated mouse (*n* = 10 mice per group). ELISA statistical calculations were performed on sera samples run in technical triplicates. Error bars indicate the standard deviation of the mean. Source data are provided as a Source Data file

Next, we performed a vaccination trial to determine the immunogenicity of a trivalent CPS8-ComP, CPS9V-ComP, and CPS14-ComP pneumococcal bioconjugate vaccine (Fig. 4a–l). Three control groups were included, one group receiving carrier protein alone (unglycosylated ComP), another group receiving a monovalent vaccine of the CPS14-ComP bioconjugate to account for IgG specificity when analyzing immune responses against other serotypes, and a third group receiving Prevnar 13^®^ as a positive control. All immunogen groups contained an equal mixture of Freund’s adjuvant, including mice receiving Prevnar 13^®^. Day 49 sera from each group were analyzed by ELISAs on plates coated with *S. pneumoniae* serotypes 8, 9V, and 14. As mentioned above, serotypes 9V and 14 are included in Prevnar 13^®^ and an elevated IgG response could be seen in Prevnar 13^®^-immunized mice against these two serotypes 49 days post vaccination. Mice receiving the monovalent CPS14-ComP bioconjugate also showed significant IgG increase specific to serotype 14 specific (Fig. 4i). Mice receiving the trivalent CPS8-/CPS9V-/CPS14-ComP bioconjugate all had statistically significant increases in serotype-specific IgG responses 49 days post vaccinations (Fig. 4j–l).

**Fig. 5:**
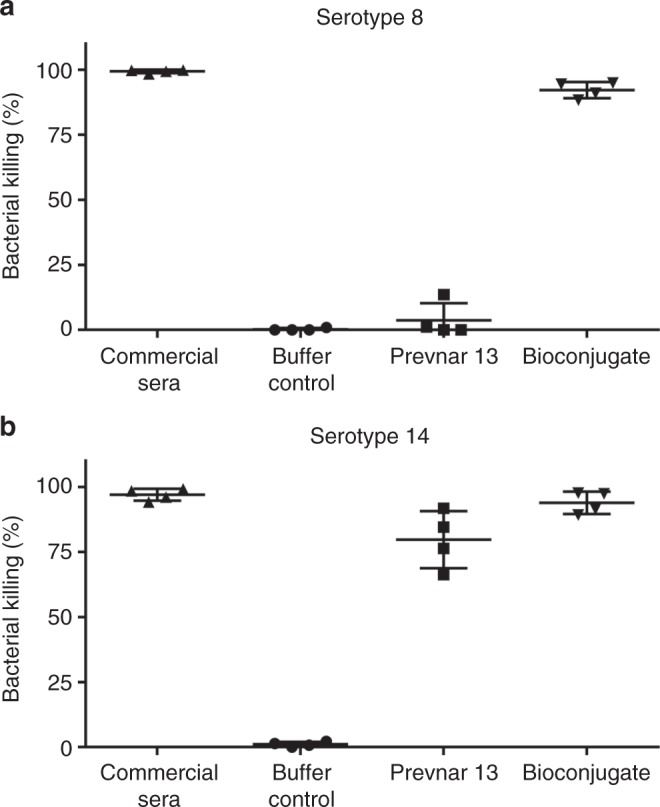
Bactericidal activity of sera from vaccinated mice against *Streptococcus* *pneumoniae* serotypes 8 and 14. Opsonophagocytosis assays (OPAs) of sera from mice vaccinated with either buffer control (*n* = two female mice), Prevnar 13^®^ (*n* = two female mice), or bioconjugate vaccine against both *S. pneumoniae* serotypes 8 (**a**) and 14 (**b**) (*n* = two female mice). OPAs were performed twice in order to have two biological replicates for interpretation. Serotype-specific commercial rabbit anti-*S. pneumoniae* sera were used as positive controls. A 5% (v v^−1^) sample serum and a bacterial multiplicity of infection (MOI) of 0.01 were added to fresh whole blood from naive mice to perform the assay. Viable bacterial counts were performed after 4 h of incubation. To determine bacterial killing, viable bacterial counts from tubes incubated with sample sera were compared to those incubated with control naive mouse sera. Results are expressed as percent bacterial killing for individual mice, with error bars representing the standard deviation of the mean. Source data are provided as a Source Data file

Because Freund’s adjuvant is not a suitable adjuvant for human clinical development, we performed another immunization trial with vaccines formulated with Imject Alum Adjuvant, a mild adjuvant containing a mixture of aluminum hydroxide and magnesium hydroxide. Vaccination cohorts included a buffer/adjuvant test group, a Prevnar 13^®^ test group, and a trivalent CPS8-/CPS9V-/CPS14-ComP bioconjugate test group. Groups of three mice were vaccinated on days 1, 14, and 28. Serum was collected on day 42 and used to determine effector functions via an opsonophagocytosis assay (OPA). Given the limited amounts of sera collected from individual mice, sera were tested for bactericidal activity against serotypes 8 and 14, as one serotype is included in Prevnar 13^®^ (serotype 14) and one is not (serotype 8). As seen in Fig. 5a, b, serum from a representative mouse vaccinated with the trivalent CPS8-/CPS9V-/CPS14-ComP bioconjugate had increased bactericidal activity against *S. pneumoniae* serotype 14 strain when compared to sera from a mock-vaccinated mouse. Importantly, that same bioconjugate vaccinated serum had high bactericidal activity against a *S. pneumoniae* serotype 8 strain, which was not observed for Prevnar 13^®^-vaccinated sera due to the absence of this conjugate in its formulation.

### Glycoengineering bioconjugates using a conventional carrier

Up to this point, we have exploited the use of ComP from *Acinetobacter baylyi* ADP1 as a carrier protein for pneumococcal bioconjugate vaccine production; however, we sought to increase the commercial applicability of this technology by engineering a conventional vaccine carrier to be compatible with our *O*-linked OTase. To this end, we generated a chimeric fusion protein consisting of the ΔE553 variant of exotoxin A from *P. aeruginosa* (EPA) C terminally fused to a ComP fragment lacking its first 28 amino acids (ComPΔ28). We used a ComP ortholog from *Acinetobacter soli* strain 110264 (accession number ENV58402) as it was most efficiently glycosylated by PglS and also found to be glycosylated at the same conserved serine as ComP from *A. baylyi* ADP1 (Supplemental Fig. 6). The EPA fusion was linked to ComPΔ28 with a glycine–glycine–glycine–serine linker and trafficked to the periplasm with a DsbA signal sequence.

**Fig. 6:**
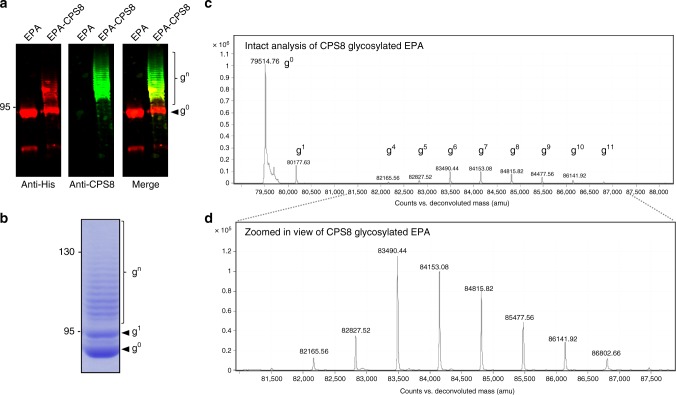
Analysis of exotoxin A from *Pseudomonas aeruginosa* (EPA) glycosylation with the CPS8 capsular polysaccharide. Western blot analysis of EPA-CPS8 bioconjugates compared against EPA alone. **a**(Left panel Anti-His channel probing for Hexa-histidine-tagged EPA purified from SDB1 expressing CPS8 in the presence or absence of PglS. **a** (Middle panel) Anti-glycan channel probing for CPS8. **a** (Right panel) Merged images for left and middle panels. **b** EPA-CPS8 separated on a SDS- polyacrylamide gel stained with Coomassie. **c**, **d** Intact protein mass spectrometry analysis showing the MS1 mass spectra for purified EPA-CPS8. The EPA fusion protein has a theoretical mass of 79,526.15Da and can be observed as the peak at 79,514.76 Da. The EPA fusion protein was also observed in multiple states of increasing mass corresponding to the CPS8 repeating subunit, which has a theoretical mass of 662Da. Varying glycoforms of the EPA-CPS8 were observed and are denoted by “g^numeric^”, where “g” stands for glycoform and the “numeric” corresponds to the number of repeating CPS8 subunits. The EPA fusion protein was modified with up to 11 repeating subunits of the CPS8 glycan. Panel d provides a zoomed in view of the varying EPA-CPS8 glycoforms. Source data are provided as a Source Data file

Because current formulations of pneumococcal conjugate vaccines do not contain a conjugate for serotype 8, we focused on generating an EPA-CPS8 pneumococcal bioconjugate. The EPA fusion was introduced into SDB1 cells co-expressing PglS and CPS8, subsequently purified, and then probed for glycosylation. As seen in Fig. 6a, b, the EPA fusion was efficiently glycosylated with CPS8 as determined by both western blot and Coomassie staining of purified glycoprotein. Furthermore, MS analysis of intact glycoproteins confirmed that the EPA fusion was repetitively modified with an increasing mass unit of 662 Da, which is the calculated mass of a single CPS8 subunit (Fig. 6c, d). The EPA fusion was found to be glycosylated with at least 11 CPS8 subunits by intact protein analysis; however, western blot and Coomassie analyses indicated that >15 subunits were able to be transferred.

**Fig. 7:**
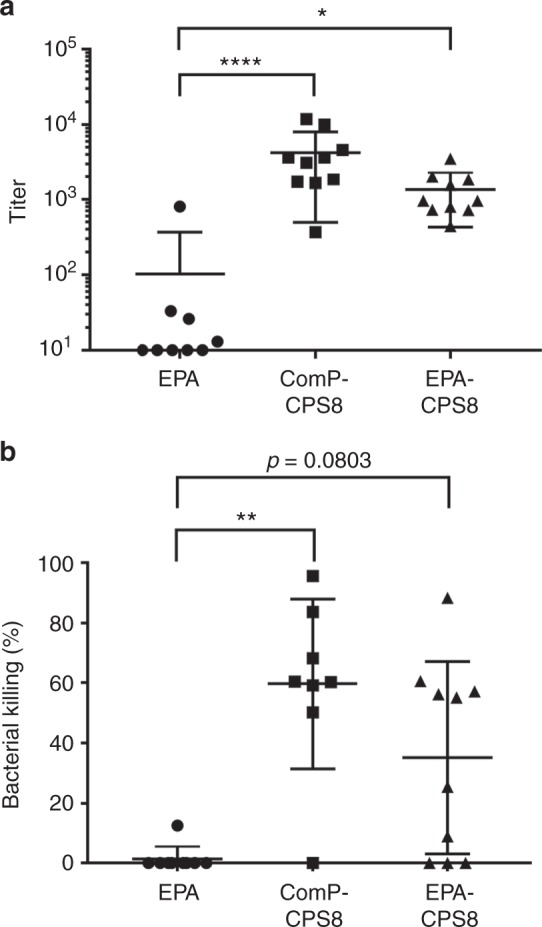
Analysis of immune responses to ComP-CPS8 and EPA-CPS8 bioconjugates in mice. **a** Titers of CPS8 IgG antibodies in mice immunized with CPS8 bioconjugate vaccines. Mouse groups were as follows: EPA (*n* = 9, mice vaccinated with 5 µg of total protein), ComP-CPS8 (*n* = 10, mice vaccinated with 5 µg total polysaccharide), and EPA-CPS8 (*n* = 10, mice vaccinated with 100 ng of total polysaccharide). All mice were immunized with 100 µL of a vaccine diluted 1:1 with Imject Alum Adjuvant on days 1, 14, and 28. Sera were collected on day 4. For the titration, enzyme-linked immunosorbent assay (ELISA) plates were coated with whole-cell serotype 8 pneumococci and incubated with 2-fold serial dilutions of sera. Each dot represents a single vaccinated mouse (*n* = 10 mice per group). ELISA statistical calculations were performed on sera samples run in technical triplicates. Titers for individual mice are shown, with error bars representing the standard error of the mean. Statistically significant titers compared to the EPA placebo group are denoted with asterisk and were determined using Kruskal–Wallis one-way analysis of variance (ANOVA). **P = 0.0223 and ****P < 0.0001. For analysis and representation purposes, negative titer values (<100) were given an arbitrary value of 10. **b** Opsonophagocytosis killing of *S. pneumoniae* serotype 8 by day 42 sera from mice immunized with ComP-CPS8 and EPA-CPS8 bioconjugate vaccines. The same mouse groups described for the IgG titers were employed for the OPA. A 40% (v v^−1^) sample of serum and bacterial multiplicity of infection (MOI) of 0.01 were added to fresh whole blood from naive mice to perform the assay. Results are expressed as percent bacterial killing for individual mice, with error bars representing the standard deviation of the mean. Statistically significant killing compared to the EPA placebo group is denoted with asterisk and were determined using Kruskal–Wallis one-way ANOVA. **P = 0.0015. Source data are provided as a Source Data file

Subsequently, we performed a vaccination experiment comparing the immunogenicity of an EPA-CPS8 pneumococcal bioconjugate to a ComP-CPS8 pneumococcal bioconjugate. Groups of 10 mice were either vaccinated with 5 µg of EPA alone (based on total protein), 5 µg of ComP-CPS8 (based on polysaccharide as determined by anthrone sulfuric acid), or 100 ng of EPA-CPS8 (based on polysaccharide as determined by MS of intact EPA-CPS8). Mice were vaccinated on days 1, 14, and 28 with serum collected on day 42. All vaccines were formulated 1:1 with imject Alum Adjuvant. ELISAs were subsequently performed to determine the IgG titers specific to CPS8. As seen in Fig. 7a, mice vaccinated with either ComP-CPS8 or EPA-CPS8 had statistically significant increases in IgG titers specific to CPS8 when compared to EPA-vaccinated mice. Additionally, the protective capacity of sera from vaccinated mice was determined using a murine adapted OPA with whole-blood leukocytes. As shown in Fig. 7b, sera from vaccinated mice immunized with ComP-CPS8 displayed high levels of bactericidal killing ranging from 84 to 50%, with one mouse not displaying any killing activity. Moreover, sera from EPA-CPS8-vaccinated mice also displayed bactericidal ranging from 88 to 10%, with three mice displaying no killing activity. Expectedly, sera from EPA-vaccinated mice did not display killing activity.

## Discussion

Traditional chemical conjugate vaccine synthesis is complex, costly, and laborious;^15^ therefore, new technologies to complement existing manufacturing pipelines are needed. One of these is bioconjugation, which has been thoroughly progressing as a feasible manufacturing alternative. The ability to glycosylate carrier proteins with polysaccharides containing Glc as the reducing end sugar has been elusive though, hindering the development of pneumococcal bioconjugate vaccines covering clinically relevant serotypes. Here we report the use of an *O-*linking OTase system for generating pneumococcal bioconjugate vaccines. Furthermore, we show that PglS naturally accepts polysaccharides containing Glc at the reducing end, a feat previously thought technically impossible due to substrate specificity limitations of all known conjugating enzymes.

The process of bioconjugation, described over a decade ago^16^, has proven to be both technically and commercially feasible. This is best evidenced by the 2015 partnership between GlaxoSmithKline and GlycoVaxyn for more than 200 million US dollars. To date, bioconjugation relies on two conjugating enzymes, PglB and PglL, with much of the focus on PglB due to its inherent ability to glycosylate soluble proteins at a known sequon^30^. As such, PglB has been the workhorse for the development and generation of bioconjugate vaccines in clinical trials. Examples include the Flexyn2a bioconjugate^18^ against *Shigella dysenteriae* as well as a tetravalent ExPEc4V bioconjugate^17^ vaccine against extraintestinal pathogenic *E. coli*. Recently, PglB was used to generate a bioconjugate vaccine against serotype 4 of pneumococcus^37^. However, serotype 4 contains *N-*acetylgalactosamine as the reducing end sugar, which is one of the known substrates for PglB.

PglL also has commercially applicable features given its ability to transfer polysaccharides with Gal at the reducing end^25^. However, until recently, PglL was thought to only glycosylate a few *Neisseria* proteins^31^, most of which were membrane-associated proteins. Research by the Wang group though has resulted in the generation of a PglL-specific sequon that can be engineered onto any carrier protein and efficiently be glycosylated by PglL, thus rendering the production of PglL-manufactured bioconjugates more practical^38,39^. However, PglB and PglL are not useful for the production of the overwhelming majority pneumococcal serotypes due to the presence of Glc at their reducing ends.

The genome of *A. baylyi* ADP1 encodes for two *O*-OTases, a PglL ortholog, which is a general OTase, and PglS, which glycosylates a single protein, ComP^28^. ComP is orthologous to type IV pilin proteins, like PilA from *P. aeruginosa* and PilE from *Neisseria gonorrhoeae*, both of which are glycosylated by the OTases TfpO (also known as PilO)^40^ and PglL (also known as PglO)^41^, respectively. TfpO glycosylates its cognate pilin at a C-terminal serine residue^33^, which is not present in ComP. Some *Acinetobacter* strains also possess TfpO orthologs^28^. PglL glycosylates PilE at an internal serine located at position 63 (ref. ^42^). ComP contains serine residues near position 63 and the surrounding residues show limited conservation to PilE from *N. gonnorrhoeae*; however, Ser 63 and its surrounding residues were not part of the ComP glycosylation site. Instead, PglS glycosylates ComP at a single serine residue located at position 84, a glycosylation site which is not a canonical LCR, rich in proline, alanine, and serine residues. The ability of PglS to transfer polysaccharides containing Glc as the reducing end sugar coupled with the identification of a previously unrecognized site of glycosylation within the pilin superfamily demonstrates that PglS is a functionally distinct OTase from PglL and TfpO, and suggests that PglS belongs to a separate family of OTases.

Using the PglS/ComP OTase/acceptor protein pair, we have generated the first polyvalent pneumococcal bioconjugate vaccine and demonstrated its immunogenicity and efficacy using correlates of protection previously established as gold standards for pneumococcal conjugate vaccines^43^. First, we demonstrate serotype-specific IgG responses of CPS8-/CPS9V-/CPS14-ComP-vaccinated mice. In these experiments, we found that the IgG response to all serotypes tested in bioconjugate-vaccinated mice were robust as determined by ELISA. Second, we showed that serum from a mouse vaccinated with pneumococcal bioconjugate vaccine was protective based on bactericidal killing assays against serotype 8 and 14 pneumococci. In addition, we have generated the first pneumococcal bioconjugate vaccine containing a conventional vaccine carrier. Namely, we have engineered the use of a ComP fragment as a glycotag, which can be added to the C terminus of EPA. We then paired the EPA fusion with the CPS8 polysaccharide and PglS, generating the EPA-CPS8 bioconjugate, a first of its kind pneumococcal bioconjugate vaccine. The EPA-CPS8 bioconjugate vaccine elicited high IgG titers specific to serotype 8 that were protective as determined via bactericidal killing. Importantly, vaccination with as little as 100 ng of polysaccharide in the EPA-CPS8 bioconjugate was able to provide protection.

Even with the introduction and implementation of pneumococcal conjugate vaccines over the past two decades, hundreds of thousands of deaths are still attributed to pneumococcus each year^10^. This is due in part to the 90+ serotypes of *S. pneumoniae* and the complex manufacturing methods required to synthesize pneumococcal conjugate vaccines. Together, these factors hinder development of broader, more protective and less costly variations of the vaccines. Our bioconjugation platform for synthesizing pneumococcal conjugate vaccines from polysaccharides with Glc at the reducing end could expedite development and lower manufacturing costs. PglS-derived bioconjugates could complement existing manufacturing pipelines or completely bypass the dependency on chemical conjugation methodologies, enabling the production of a more comprehensive pneumococcal conjugate vaccine. Here we present data using the natural acceptor protein, ComP, as well as a proof-of-principle EPA fusion protein as the targets of PglS glycosylation. However, future iterations of the EPA vaccine construct will impart additional sites of glycosylation to increase the glycan to protein ratio as well as expand upon the number of serotypes in order to develop a comprehensive pneumococcal bioconjugate vaccine. Regardless, we present compelling data indicating that these pneumococcal bioconjugates have the potential for further commercial development. Importantly, the platform technology we present in this study is not limited to pneumococcal polysaccharides, but in fact, has vast applicability for generating bioconjugate vaccines for many important human and animal pathogens that are incompatible with PglB and PglL. Notable examples include the human pathogens *Klebsiella pneumoniae* and Group B *Streptococcus* as well as the swine pathogen *Streptococcus suis*, all immensely relevant pathogens with no licensed vaccines available.

## Methods

### Bacterial strains, plasmids, and growth condition

Strains and plasmids used in this work are listed in Supplemental Table 1. Unless otherwise stated, *E. coli* strains were grown in Terrific Broth (TB) at 37 °C overnight for ComP glycoprotein production or Super Optimal Broth (SOB) at 30 °C overnight for EPA glycoprotein production. *Streptococcus pneumoniae* strains were grown in brain heart infusion (BHI) broth or sheep blood agar plates at 37 °C in 5% CO_2._ For plasmid selection, the antibiotics were used at the following concentrations: ampicillin (100 μg mL^−1^), tetracycline (20 μg mL^−1^), chloramphenicol (12.5 μg mL^−1^), kanamycin (20 μg mL^−1^), and spectinomycin (80 μg mL^−1^) were added as needed. Oligonucleotides used in this study are listed in Supplementary Table 2.

### Heterologous glycosylation in *E. coli*

*Escherichia coli* SDB1 was made electrocompetent by growing cells to mid-logarithmic stage followed by two rounds of washing in 10% glycerol and a final resuspension in 1/250th of the original culture volume. Cells were electroporated with plasmids encoding the glycan synthesis loci, acceptor proteins, and OTases. Colonies were picked and grown at 37 °C in TB or SOB with appropriate antibiotic selection and immediately induced with 0.05–0.1 mM isopropyl β-d-1-thiogalactopyranoside or 0.2% arabinose as needed and left overnight at 37 °C. Cultures requiring arabinose induction received a second dose of arabinose after 4 h. Cell pellets were obtained at stationary phases and prepared for western blot analysis.

### Western blotting

Cell lysates containing the equivalent of OD_600_ = 0.1 units were loaded on 12.5% or 7% in-house prepared sodium dodecyl sulfate-polyacrylamide gel electrophoresis (SDS-PAGE) gels, which were then transferred to nitrocellulose membranes (Bio-Rad). Western blots were employed to determine protein modification. Primary antibodies included Pneumococcus Type 8 Serum (Ref. # 16751), Pneumococcus Type 9 Serum (Ref. # 16903), and Pneumococcus Type 14 Serum (Ref. # 16751), all used at 1:1000 dilutions. Additional antibodies included 6×-His Tag Monoclonal Antibody (HIS.H8) (Catalog # MA1-21315), used at 1:1000, and anti-Pseudomonas exotoxin A antibody (P2318-1ML), used at 1:5000. Secondary antibodies included Licor IRDye 680RD goat anti-mouse (925-68070) and goat anti-rabbit 800CW (926-32211) used at 1:10,000 dilutions. Western blotting was performed according to our previously published protocols^28^. Briefly, samples were separated by SDS-PAGE, transferred to nitrocellulose, blocked with Licor TBS blocking buffer, incubated with primary antibodies for 30 min, washed three times in TBS supplemented with Tween-20, incubated with secondary antibodies for 30 min, washed three times with TBS supplemented with Tween-20, and then visualized using an Odyssey Infrared Imaging System (LiCor Biosciences, USA).

### Purification of proteins and glycoproteins

C terminally Hexa-histidine-tagged ComP and ComP bioconjugates were purified from *E. coli* total membrane preparations. Cells were grown overnight in 2 L of TB at 37 °C, washed with phosphate-buffered saline (PBS) buffer, and re-suspended in 60 mL of the same buffer. Cells were lysed by two rounds of cell disruption at approximately 20 kPSI using a French press (Aminco), followed by the addition of a protease inhibitor cocktail (Roche). Lysates were centrifuged twice for 30 min at 20,000 × *g* to pellet cell debris. Supernatants were ultra-centrifuged at 100,000 × *g* for 60 mins to pellet total membranes. The pellets were re-suspended in PBS buffer containing 0.5% *n*-dodecyl-β-d-maltoside (DDM) and membrane proteins were solubilized by tumbling for 48 h. An equal volume of PBS was added to the suspension to reduce detergent concentration to 0.25% and the suspension was ultra-centrifuged at 100,000 × *g* for 60 mins. Solubilized membranes were filtered through 0.45 and 0.22 μm filters and loaded on a His-Trap HP column (GE Healthcare) fitted to an ÄKTA purifier (Amersham Biosciences, Sweden). The column was equilibrated with a PBS/DDM buffer containing 20 mM imidazole prior to loading the sample. Unbound proteins were removed by washing the column with seven column volumes of buffer containing 20 and 30 mM imidazole in PBS stepwise. To elute proteins bound to the column, a gradient elution with an incremental increase in imidazole concentration was used. The majority of unconjugated and conjugated ComP eluted between 180 and 250 mM imidazole. Imidazole was removed by an overnight round of dialysis followed by two 2-h rounds through a 3.5 kDa dialysis membrane (Spectrum labs) in a 250 mL dialysis buffer composed of PBS containing 0.25% (w v^−1^) DDM. The final theoretical concentration of imidazole post dialysis was about 0.007 mM. Proteins were quantified using a DC kit (Bio-Rad), after which the samples were diluted to the appropriate concentrations for mouse immunizations.

C terminally Hexa-histidine-tagged EPA fusion proteins were purified from *E. coli* lysates lysed using mechanical disruption at 35,000 PSI using a cell disruptor from Constant Systems. Lysates were clarified at 15,000 × *g* for 30 min. The supernatents were passed over 3 mL of nickel NTA agarose, washed with 10 column volumes of buffer containing 20 mM Tris, 10 mM imidazole, 500 mM NaCl, pH 8.0, and eluted with the same buffer containing 300 mM imidazole. Eluted proteins were concentrated using an Amicon Ultra-15 concentrator, centrifuged at 10,000 × *g* for 10 min, and polished on a superdex 200 size exclusion column. Fractions enriched for EPA proteins pooled, concentrated, and buffer exchanged into PBS. Proteins were quantified using a DC kit for total protein concentrations and a modified anthrone sulfuric method for carbohydrate estimation. Briefly, to 100 μL of sample, 4 mL of a 2 mg mL^−1^ anthrone-concentrated sulfuric acid solution was rapidly added. The sample was vortexed and heated in boiling water bath for 10 min. Aliquots were measured for absorbance at 620 nm. Carbohydrate estimations were based off a standard curve of the type 8 pneumococcal CPS from ATCC (cat. # ATCC 20-X).

### Murine model immunizations

All murine immunizations complied with all relevant ethical regulations for animal testing and research. Immunizations were conducted at the Southern Alberta Cancer Research Institute antibody services and Washington University School of Medicine in St. Louis according to institutional guidelines and received approval from the University of Calgary Animal Research and Education Executive Committee and the Institutional Animal Care and Use Committee at Washington University in St. Louis, respectively. For the CPS14-ComP monovalent immunization, 4–6-week-old female BALB/c mice were injected with 50 μL of purified protein/glycoprotein (3 µg total protein) with 50 μL of Freund’s adjuvant. Two groups of mice (*n* = 10) were injected with either unglycosylated ComP (placebo) or CPS14-ComP bioconjugate. Sera from the mice were obtained before immunizations and 7, 21, 35, and 49 days post immunizations. Booster doses were given on days 14 and 28. The same procedure was followed for the trivalent immunization, except four groups of mice (*n* = 10) were used for the four different immunization groups. These groups were injected with 100 µL containing 3 µg of unconjugated ComP (placebo) and Freund’s adjuvant, 100 µL containing 3 µg of ComP-CPS14 conjugate and Freund’s adjuvant, 100 µL containing 9 µg of a glycoprotein mixture (ComP-CPS8, ComP-CPS9V, and ComP-CPS14) and Freund’s adjuvant, or 100 µL of a 1:10 diluted stock of Prevnar 13^®^ and Freund’s adjuvant. CPS-ComP bioconjugates were formulated by total protein for this immunization.

Another trivalent immunization experiment was conducted with groups of three 4–6-week-old female BALB/c mice. Each immunization group was subcutaneously injected with 100µL of a 1:1 immunogen (3 µg of protein of each of the trivalent bioconjugate or a 1:10 diluted stock of Prevnar 13^®^) to Imject Alum Adjuvant. Mice were vaccinated on days 0, 14, and 28 and then sacrificed on day 42 for sera collection.

A fourth immunization experiment was conducted with groups of three 4–6-week-old BALB/c mice (five female and five male per group). Mice were immunized subcutaneously with 100 µL of EPA (5 µg total protein), 100 µL of ComP-CPS8 (5 µg total polysaccharide), or 100 µL of EPA-CPS8 (0.1 µg total polysaccharide) on days 0, 14, and 28 and then sacrificed on day 42 for sera collection. Vaccines were formulated 1:1 with Imject Alum Adjuvant.

### Enzyme-linked immunosorbent assays

*Streptococcus pneumoniae* strains grown overnight in BHI broth at 37 °C in 5% CO_2_ were washed in PBS and the optical density was adjusted to OD_600_ = 0.6 units. Cells were heat inactivated at 60 °C for 2–4 h followed by immobilization on high binding 96-well plates (Corning) by adding 50 μL per well. Plates were incubated on a tumbler overnight at 4 °C. The following day, wells were washed three times with PBST (phosphate-buffered saline-Tween) (100 μL per well) before blocking with 5% skimmed milk (250 μL per well) for 2 h. The wells were washed three times with PBST. Plates were incubated for 1 h at room temperature with mouse sera (100 μL per well) at a 1:500 dilution in 2.5% skimmed milk in PBST for assessing immunogenicity of ComP bioconjugates. For the positive control, commercial rabbit polyclonal antibodies against CPS were used (catalog numbers described above). Negative control wells were treated with skimmed milk without any primary antibody. After incubation with the primary antibody, wells were washed three times with PBST followed by a 1 h incubation with secondary HRP-conjugated anti-mouse IgG (Cat. # 7076) diluted 1:4000 (100 μL per well) in 2.5% skimmed milk in PBST. After incubation, the wells were washed three times with PBST and 100 μL of the chromogenic substrate TMB (Cell Signaling Technology) was added to each well. Plates were incubated at room temperature for 5 min, after which the absorbance at 650 nm was measured using a BioTek™ plate reader.

For IgG titer determinations, ELISA plates were coated with 100 µL of 1 × 10^8^ CFU mL^−1^ of *S. pneumoniae* serotype 8 grown approximately to mid-log phase. Bacteria were washed twice in PBS and suspended in water prior to coating. ELISA plates were allowed to air dry in a biological hood for 24 h. Fifty microliters of methanol were then added to each well and allowed to air dry. Plates were stored in a re-sealable bag protected from the light until use. To perform the titration of mouse total IgG antibodies, day 42 sera was serially diluted (2-fold) in PBST and antibodies were detected using an anti-mouse, HRP-linked IgG (Cell Signaling Technology # 7076) diluted 1:4000. For mouse serum titrations, the reciprocal of the last serum dilution that resulted in an optical density at 450 nm equal to or lower than 0.2 was considered the titer of that serum. For representation purposes, negative titers (less than or equal to the cutoff) were given an arbitrary titer value of 10. Inter-plate variations were controlled by including an internal reference positive control on each plate. This control was hyper-immune sera from a mouse previously immunized with the ComP-CPS8 bioconjugate vaccine. The ELISA reactions in TMB were stopped when an OD450nm of ~1 was obtained for the internal positive control.

### Site-directed mutagenesis

Mutagenic primers were designed using Primer X, a web-based primer design program (http://www.bioinformatics.org/primerx/). Primers used are listed in Supplemental Table 2. PCR reactions were performed using *Pfu* polymerase and 2–10 ng of pMN2 as template. The PCR reaction consisted of an initial denaturation of 30 s at 95 °C followed by 16 cycles of 30 s at 95 °C, 60 s at 55 °C, 360 s at 68 °C with no final extension. PCR reactions were *Dpn*I digested for 2 h to remove the template plasmid, then transformed into electrocompetent DH5α cells, and grown on ampicillin for plasmid selection. Colonies were sequenced to confirm mutagenesis.

### Digestion of ComP-CPS14 conjugate

CPS14-ComP was affinity purified and separated via SDS-PAGE and Coomassie stained. SDS-PAGE separated CPS14-ComP bands were excised and destained in a 50:50 solution of 50 mM NH_4_HCO_3_:100% ethanol for 20 min at room temperature with shaking at 750 rpm. Destained bands were then washed with 100% ethanol, vacuum dried for 20 min, and rehydrated in 10 mM dithiothreitol (DTT) in 50 mM NH_4_HCO_3_. Reduction was carried out for 60 min at 56 °C with shaking. The reducing buffer was then removed and the gel bands washed twice in 100% ethanol for 10 min to ensure the removal of remaining DTT. Reduced ethanol washed samples were sequentially alkylated with 55 mM iodoacetamide in 50 mM NH_4_HCO_3_ in the dark for 45 min at room temperature. Alkylated samples were then washed with two rounds of Milli-Q water and 100% ethanol then vacuum dried. Alkylated samples were then rehydrated with 10 ng µl^−1^ GluC (Promega, Madison, WI, USA) in 40 mM NH_4_HCO_3_ at 4 °C for 1 h. Excess GluC was removed, gel pieces were covered in 40 mM NH_4_HCO_3_, and incubated for 24 h at 37 °C. Peptides were concentrated and desalted using C_18_ stage tips^44,45^ and stored on tip at 4 °C. Peptides were eluted in Buffer B (0.5% acetic acid, 80% MeCN) and dried before analysis by liquid chromatography-mass spectrometry (LC-MS).

### Reversed phase LC-MS and HCD MS-MS

Purified peptides were re-suspended in Buffer A* and separated using an in-house packaged 25 cm, 75 µm inner diameter, 360 μm outer diameter, 1.7 µm 130 Å CSH C_18_ (Waters, Manchester, UK) reverse-phase analytical column with an integrated HF-etched nESI tip. Samples were loaded directly onto the column using an ACQUITY UPLC M-Class System (Waters) at 600nLmin^−1^ for 20 min with Buffer A (0.1% formic acid (FA)) and eluted at 300nL min^−1^ using a gradient altering the concentration of Buffer B (99.9% acetonitrile, 0.1% FA) from 2 to 32% B over 60 min, then from 32 to 40% B in the next 10 min, then increased to 80% B over 8 min period, held at 100% B for 2 min, and then dropped to 2% B for another 10 min. Reverse-phase separated peptides were infused into a Q-Exactive (Thermo Scientific) mass spectrometer and data acquired using data-dependent acquisition. Two methods were used to identify putative glycopeptides. Method A aimed to enable robust peptide identification in which one full precursor scan (resolution 70,000; 350–1850*m*/*z*, AGC target of 1 × 10^6^) was followed by 10 data-dependent higher energy collisional dissociation (HCD) MS-MS events (resolution 35k AGC target of 1 × 10^5^ with a maximum injection time of 110 ms, NCE 26 with 25% stepping) with 90 s dynamic exclusion enabled. Method B aimed to enable more complete characterization of glycans within glycopeptides with one full precursor scan (resolution 70,000; 350–1850*m*/*z*, AGC target of 1 × 10^6^) followed by 10 data-dependent HCD MS-MS events (resolution 35k AGC target of 5 × 10^5^ with a maximum injection time of 250 ms, NCE 13 with 25% stepping) with 90 s dynamic exclusion enabled.

### Database interrogation of identified glycopeptides

Raw files were processed manually to identify potential glycopeptides based on the diagnostic oxonium 204.08*m/z* ion. Putative glycopeptide-derived scans were manually inspected and identified as possible GluC-derived ComP glycopeptides based on the presence of an intense deglycosylated ComP-derived peptide ion, matching within 10 ppm using the Expasy FindPept tool (https://web.expasy.org/findpept/). To facilitate peptide assignments, the resulting glycopeptides was manually annotated according to ref. ^46^ with the aid of the Protein Prospector tool MS-Product (http://prospector.ucsf.edu/prospector/cgi-bin/msform.cgi?form = msproduct).

### Intact protein analysis

Intact analysis was performed using a 6520 Accurate Mass Quadrupole Time-of-Flight mass spectrometer (Agilent, Santa Clara, CA, USA). Protein samples were re-suspended in 2% ​ACN, 0.1% ​trifluoroacetic acid, and immediately loaded onto a C5 Jupiter 5 μm 300 Å 50 mm × 2.1 mm column (Phenomenex, Torrance, CA, USA) using an Agilent 1200. Samples were desalted by washing with buffer A (2% ​ACN, 0.1% FA) for 4 min and then separated with a 12 min linear gradient from 2 to 100% buffer B (80% ​ACN, 0.1% FA) at a flow rate of 0.200 mL min^−1^. MS1 mass spectra were acquired at 1 Hz between a mass range of 300–3000*m/z*. Intact mass analysis and deconvolution was performed using MassHunter B.06.00 (Agilent).

### Opsonophagocytosis assay

The assays were performed as previously described^47,48^ and are briefly described below. *Blood collection*. Blood was collected by intracardiac puncture from naive female mice (Charles River, Wilmington, MA, USA), treated with sodium heparin, then diluted to obtain 6.25 × 10^6^ leukocytes mL^−1^ in RPMI-1640 supplemented with 5% heat-inactivated fetal bovine serum, 10 mM HEPES, 2mM l-glutamine, and 50 μM 2-mercaptoethanol. All reagents were from Gibco (Invitrogen, Burlington, ON, Canada). *Bacterial suspension preparation*. Isolated colonies on sheep blood agar plates of either *S. pneumoniae* serotypes 8 or 14 (Statens Serum Institut, Denmark) were inoculated in 5 mL of Todd–Hewitt Broth (THB) (Oxoid, Thermo Fisher Scientific, Nepean, Canada) and incubated for 16 h at 37 °C with 5% CO_2_. Working cultures were prepared by transferring 0.1 mL of 16 h cultures into 10 mL of THB, which was then incubated for 5 h. Bacteria were washed three times and re-suspended in PBS to obtain an OD_600_ value of 0.6, which corresponds to 1.5 × 10^8^ and colony-forming units (CFU)mL^−1^ and to 3.5 × 10^8^CFUmL^−1^ for serotype 8 and serotype 14, respectively. Final bacterial suspensions were prepared in complete cell culture medium to obtain a concentration of 6.25 × 10^4^CFUmL^−1^. The number of CFUmL^−1^ in the final suspensions was determined by plating samples onto Todd–Hewitt agar (THA). *Opsonophagocytosis Assay*. Diluted whole blood (5 × 10^5^ total leukocytes) was mixed with 5 × 10^3^ CFU of *S. pneumoniae* serotype 8 or 14 (multiplicity of infection of 0.01) and 5% (v v^−1^) of serum from control (placebo) or vaccinated mice in a microtube to a final volume of 0.2 mL. Microtubes were incubated for 4 h at 37 °C with 5% CO_2_, with shaking. After incubation, viable bacterial counts were performed on THA. Tubes with the addition of naive mouse sera or commercial rabbit anti-*S. pneumoniae* types 8 or 14 serum (Statens Serum Institut, Denmark) were used as negative and positive controls, respectively. The percentage of bacterial killing was determined using the following formula: percent bacteria killed = [1 − (bacteria recovered from sample tubes/bacteria recovered from negative control tubes with naive sera)] × 100.

### Reporting summary

Further information on experimental design is available in the Nature Research Reporting Summary linked to this article.

## Supplementary information

Supplementary Information

Reporting Summary

## Data Availability

The authors declare that data supporting the findings of this study are available within the paper and its supplemental files. The source data underlying Figs. 1, 3, 4, 5, 6, 7 and supplemental Figs. 2 and 5 are provided as a Source Data file.
