# Supplementary material for: A platform for glycoengineering a polyvalent pneumococcal bioconjugate vaccine using E. coli as a host
Source: Nat Commun. 2019 Feb 21;10:891. doi: 10.1038/s41467-019-08869-9 (PMC6385209; doi:10.1038/s41467-019-08869-9)
Supplement: Supplementary file 1 — Supplementary Information [file 41467_2019_8869_MOESM1_ESM.pdf]

Supplemental Files

A platform for glycoengineering a polyvalent pneumococcal bioconjugate vaccine using *E. coli* as a host

Harding et al.

Supplementary Figure 1

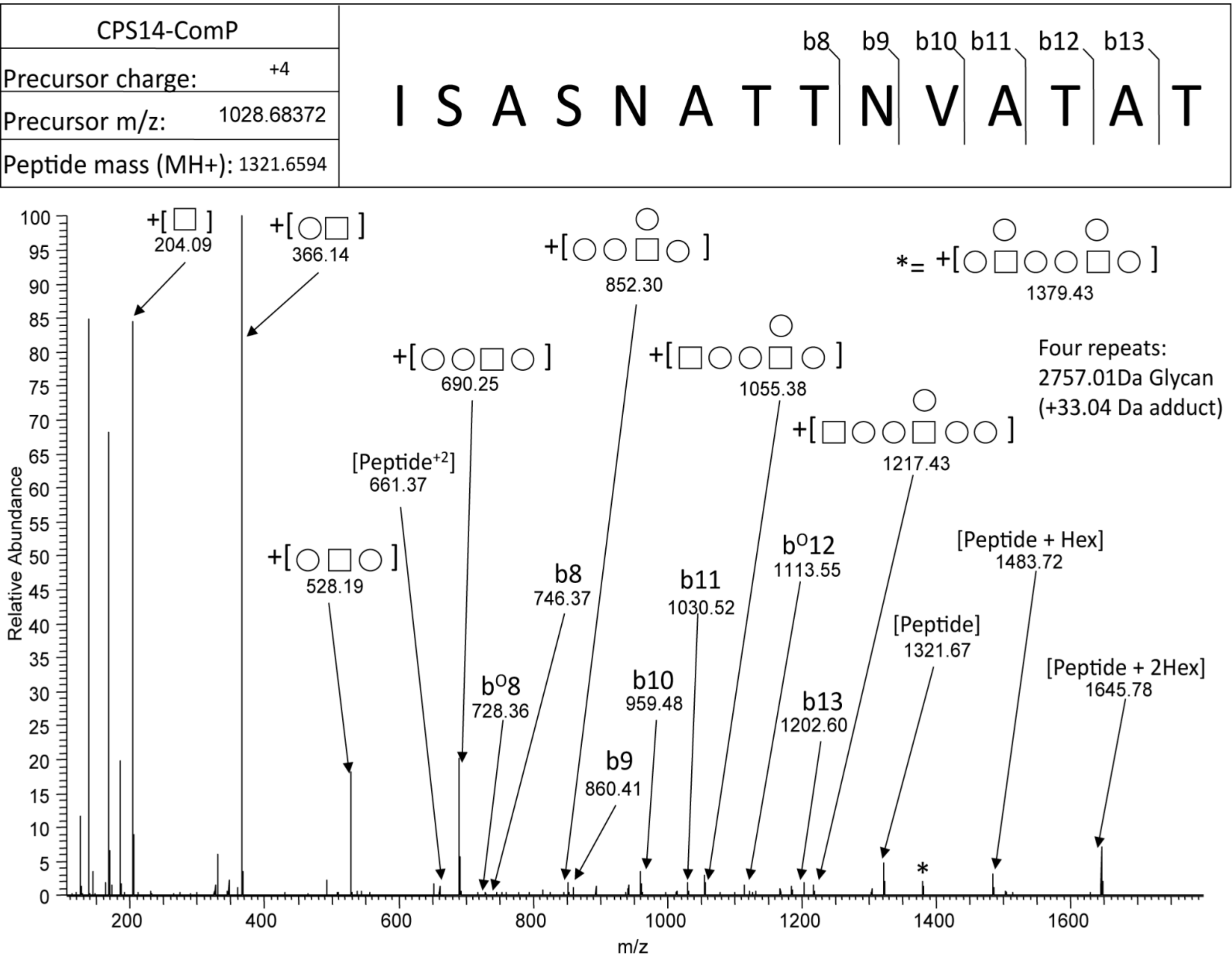

Higher energy collisional dissociation (HCD) fragmentation spectra of GluC digested CPS14-ComP bioconjugates. GluC digested CPS14-ComP was subjected to HCD fragmentation enabling the confirmation of a semi-GluC derived single peptide attached to a glycan with the CPS14 repeating subunit. Additional glycopeptides were also observed decorated with extended glycans corresponding to up to four tetrasaccharide repeat units.

Supplementary Figure 2

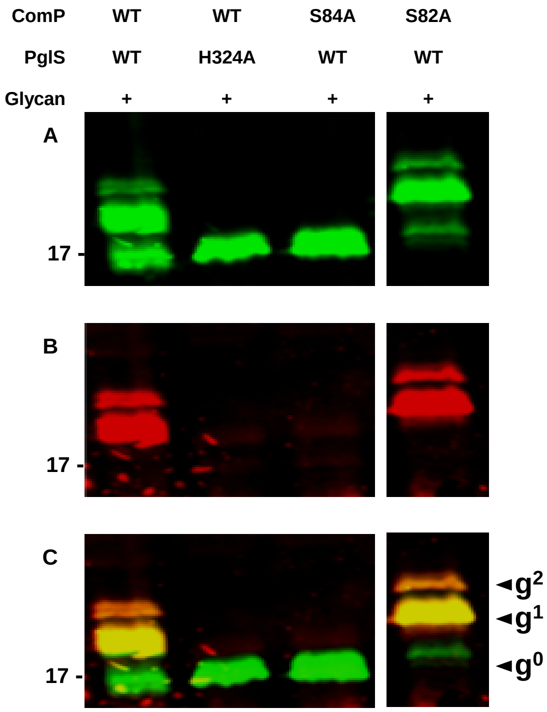

Point mutational analysis of ComP confirms serine 84 is the likely site of glycosylation. Serine 84 of ComP was mutated to an alanine and probed for glycosylation. *(A-C)* SDB1 cells expressing either WT ComP or ComP[S84A] in the presence of PglS and the *C. jejuni* heptasaccharide were probed via western blotting for protein glycosylation. As a negative control, the inactive PglS[H324A] variant was co-expressed with WT ComP and the *C. jejuni* heptasaccharide. *(A)* Anti-His channel probing for ComP expression and glycosylation. *(B)* Anti-glycan channel probing for the *C. jejuni* heptasaccharide. *(C)* Merged image for panels *A* and *B*. Source data are provided as a Source Data file.

Supplementary Figure 3

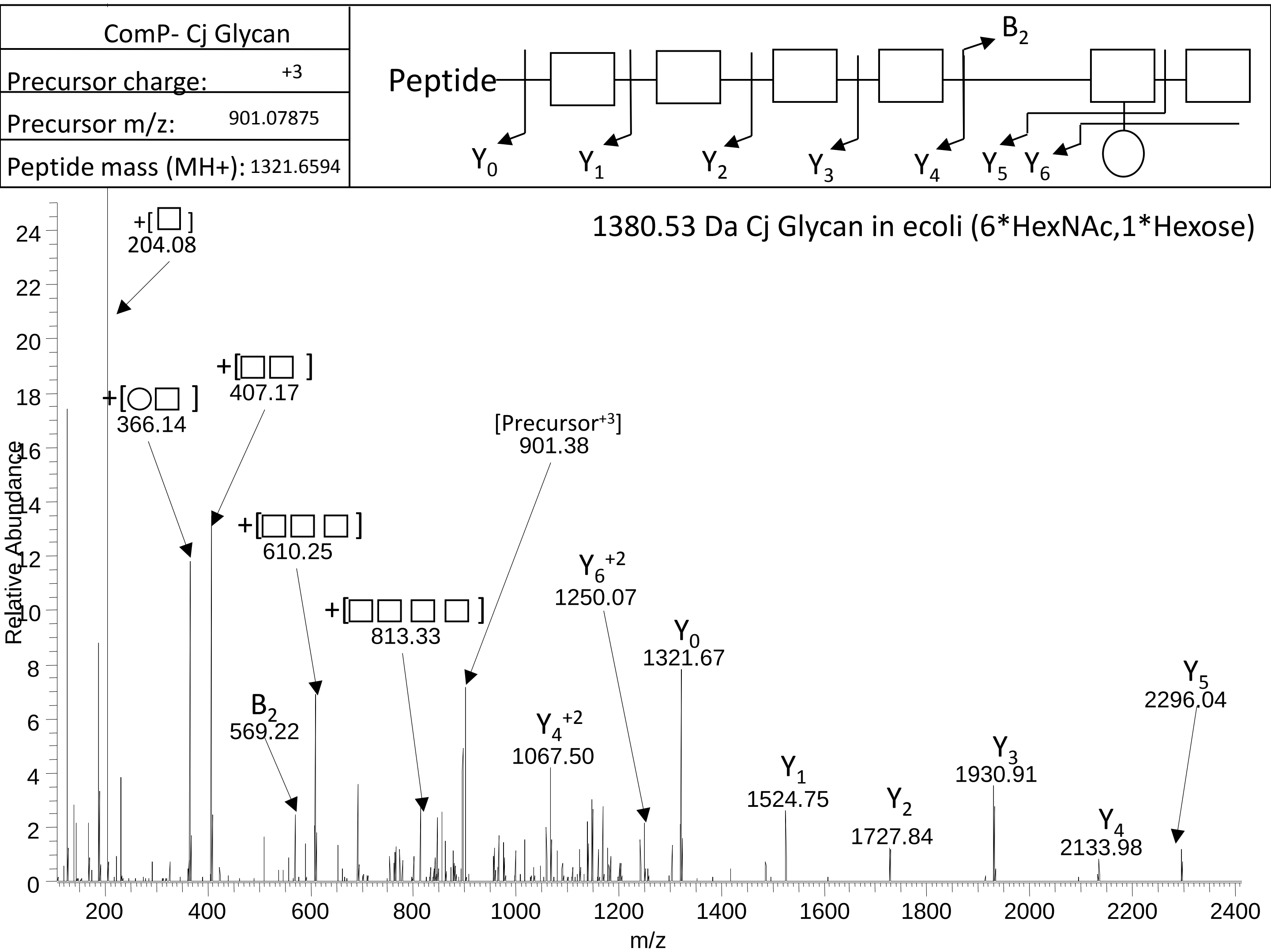

Higher energy collisional dissociation (HCD) fragmentation spectra of GluC digested ComP glycosylated with the *C. jejuni* heptasaccharide (ComP-Glycan$_{Cj}$). GluC digested ComP-Glycan$_{Cj}$ was subjected to HCD fragmentation enabling the confirmation of a single peptide attached to a glycan with the CPS14 repeating subunit. Low collision energies regimes were undertaken to confirm the glycosylation of the peptide ISASNATTNVATAT with a 1380.53 Da glycan corresponding to 6*HexNAc,1*Hexose.

Supplementary Figure 4

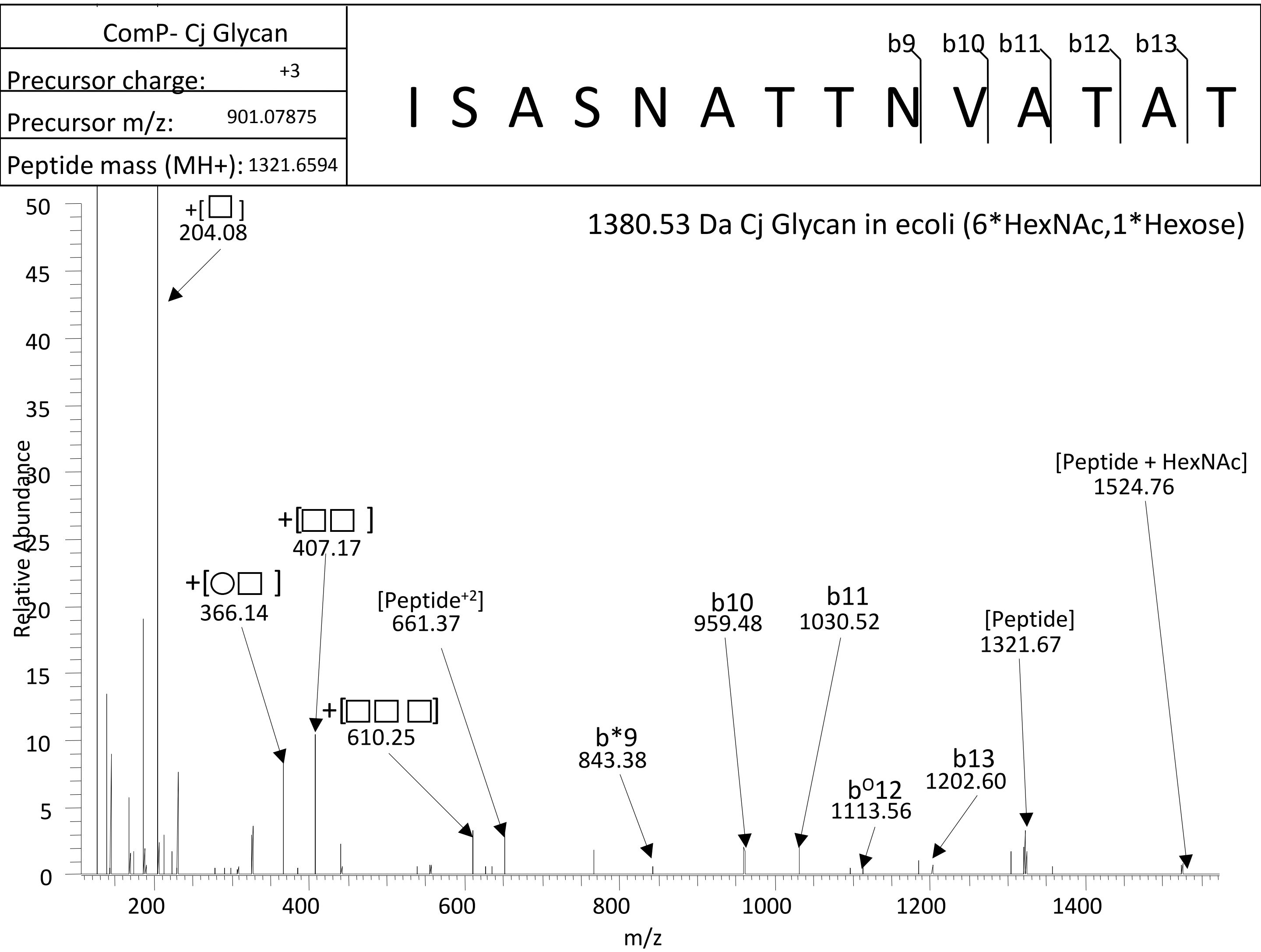

Higher energy collisional dissociation (HCD) fragmentation spectra of GluC digested ComP glycosylated with the *C. jejuni* heptasaccharide (ComP-Glycan$_{Cj}$). GluC digested ComP-Glycan$_{Cj}$ was subjected to HCD fragmentation enabling the confirmation of a single peptide attached to a glycan with the CPS14 repeating subunit. High collision energies regimes were undertaken to confirm the glycosylation of the peptide ISASNATTNVATAT with a 1380.53 Da glycan corresponding to 6*HexNAc,1*Hexose.

Supplementary Figure 5

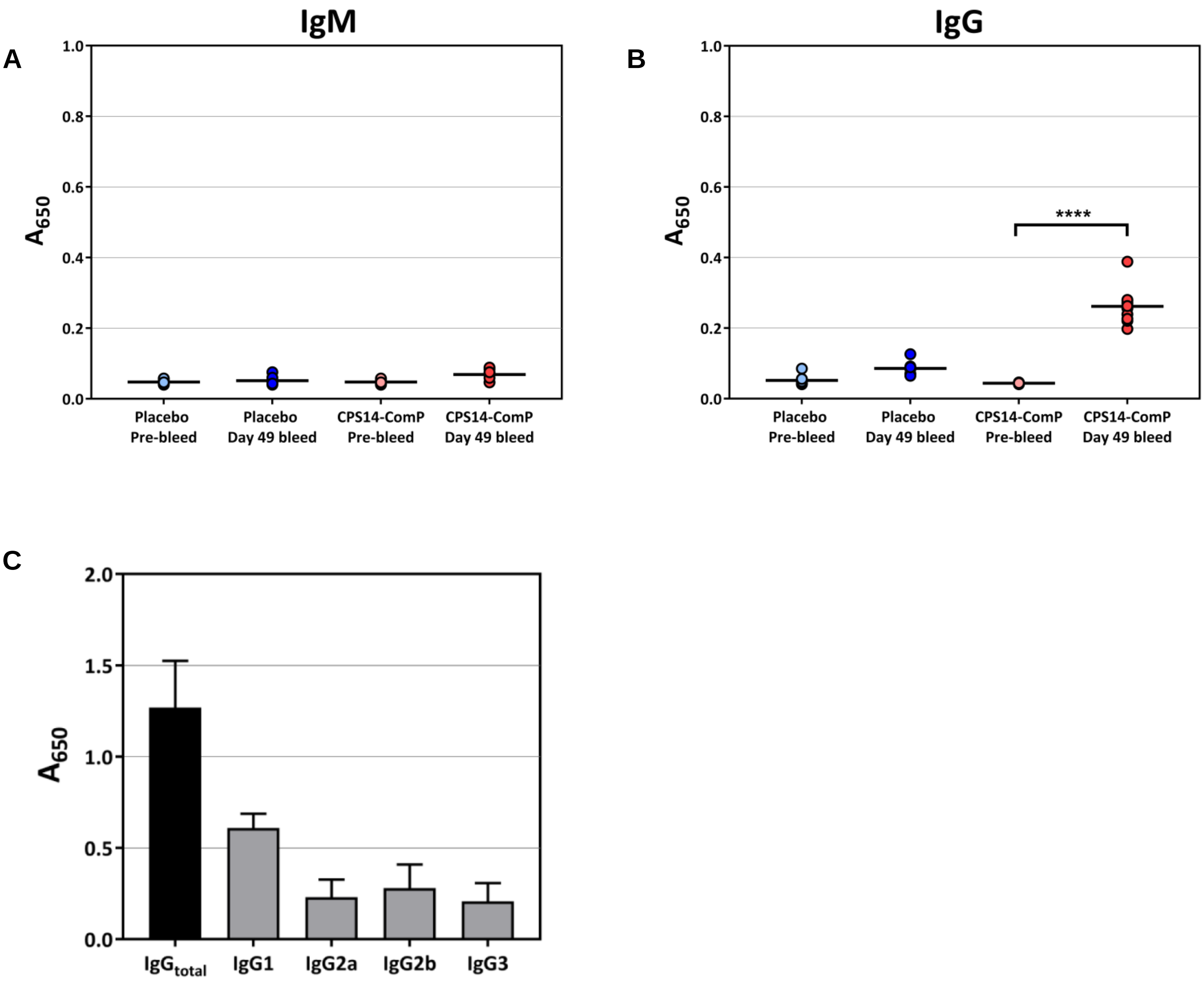

Antibody responses towards monovalent CPS14-ComP bioconjugate vaccination. Sera from placebo vaccinated and CPS14-ComP vaccinated mice were used to probe for IgM *(A)* and IgG *(B)* antibody responses measured against heat killed whole cell *S. pneumoniae* serotype 14. The Rout method for identifying outliers was performed and used to remove two data points from the IgG Placebo Day 49 bleed group as well as two points from the IgG CPS14-ComP Pre-bleed group. A Kruskal-Wallis test was subsequently performed and demonstrated that a statistically significant increase in serotype 14 specific IgG titers was observed (**** p= 0.0001). (*C)* IgG subclass responses in mice vaccinated with the CPS14-ComP bioconjugate. Sera from CPS14-ComP vaccinated mice were used for an ELISA probing for IgG subclasses against heat killed whole cell *S. pneumoniae* serotype 14. Kruskal-Wallis analysis a statistically significant increase of IgG1 subtype over IgG2a (* p=0.0166) and IgG3 (** p=0.0080). Each dot represents a single vaccinated mouse. Error bars indicate the standard deviation of the mean. Source data are available as a Source Data file.

Supplementary Figure 6

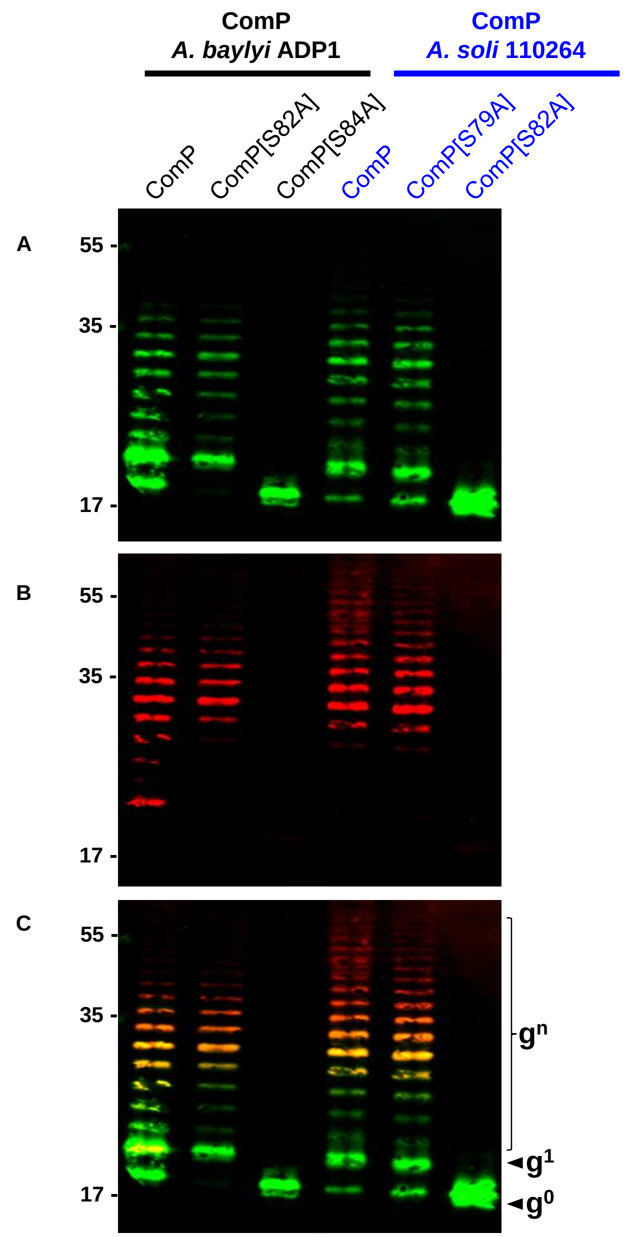

A conserved and homologous serine is the likely site of glycosylation in ComP proteins from *A. baylyi* ADP1 and *A. soli* 110264. Serines 82 and 84 of $ComP_{ADP1}$ and the homologous serines 79 and 82 of $ComP_{110264}$ were mutated to an alanine and probed for glycosylation in the presence of PglS and the serotype 8 capsular polysaccharide. *(A-C)* SDB1 cells expressing ComP variants in the presence of PglS and CPS8 were probed via western blotting for protein glycosylation. *(A)* Anti-His channel probing for ComP expression and glycosylation. *(B)* Anti-glycan channel probing for CPS8. *(C)* Merged image for panels *A* and *B*.

Supplementary Table 1

Strains and Plasmids	Description	Reference/ Source
Strains		
E. coli SDB1	W3110, Δ*waaL* ligase, Δ*wecA* glycosyltransferase	1
E. coli DH5α	General cloning strain	Invitrogen
S. pneumoniae serotype 8, 9V, and 14	Wild type pneumococci strains expressing either the serotype 8, 9V, or 14 capsular polysaccharides	Statens Serum Institut
Plasmids		
pEXT20	Cloning vector, Amp^R, IPTG inducible	2
pMN1	6X His-tagged $ComP_{ADP1}$ cloned in BamHI and SalI sites of pEXT20, Amp^R, IPTG inducible	This work
pMN2	Non-coding region and $PglS_{ADP1}$ cloned in SalI and PstI sites of pMN1, Amp^R, IPTG inducible	This work
pMN4	$PglS_{ADP1}$[H324A] in pMN2 background	This work
pMN9	$ComP_{ADP1}$[S82A] mutant of pMN2	This work
pMN10	$ComP_{ADP1}$[S84A] mutant of pMN2	This work
pMAF10	HA-tagged PglB cloned in pMLBAD, TpR, Arabinose inducible	3
pAMF10	C-10× His-tagged NmPglL cloned into pEXT20, AmpR, IPTG inducible	4
pIH18	C-6X His-tagged AcrA from *C. jejuni* cloned into pEXT21, SpR, IPTG inducible	5
pAMF22	C-6X His-tagged dsbA1 from *N. meningitidis* MC58 cloned into pMLBAD, Tp^R Arabinose inducible	4
pACYCpglBmut	pACYC184-based plasmid encoding the *C. jejuni* pgl locus with mutations W458A and D459A in PglB. Cm^R, IPTG inducible.	3
pNLP80	*S. pneumoniae* CPS14 cluster on pWSK129, Kan^R	6
pB-8	*S. pneumoniae* CPS8 cluster on pBBR1MCS-3, Tc^R	7
pWKS130-9V	*S. pneumoniae* CPS9V cluster on pWKS130, Kan^R	This work
pCH1	6X His-tagged $ComP_{110264}$ cloned into pEXT20, Amp^R, IPTG inducible	This work
pCH2	$ComP_{110264}$[S79A] mutant of pCH1	This work
pCH3	$ComP_{110264}$[S82A] mutant of pCH1	This work
pCH4	pEXT20 containing the ΔE553 variant of exotoxin A of *P. aeruginosa* (EPA) with an N-terminal DsbA signal sequence and a C-terminal glycotag consisting of $ComP_{110264}$ without the first 28 amino acids. A peptide linker (GGGS) fuses EPA to the ComP fragment.	This work

[1]Garcia-Quintanilla, F., Iwashkiw, J. A., Price, N. L., Stratilo, C. & Feldman, M. F. Production of a recombinant vaccine candidate against *Burkholderia pseudomallei* exploiting the bacterial *N*-glycosylation machinery. *Front Microbiol* **5**, 381, doi:10.3389/fmicb.2014.00381 (2014).
[2]Dykxhoorn, D. M., St Pierre, R. & Linn, T. A set of compatible tac promoter expression vectors. *Gene* **177**, 133-136 (1996).
[3]Feldman, M. F. *et al.* Engineering *N*-linked protein glycosylation with diverse O antigen lipopolysaccharide structures in *Escherichia coli*. *Proc Natl Acad Sci U S A* **102**, 3016-3021, doi:10.1073/pnas.0500044102 (2005).
[4]Faridmoayer, A., Fentabil, M. A., Mills, D. C., Klassen, J. S. & Feldman, M. F. Functional characterization of bacterial oligosaccharyltransferases involved in *O*-linked protein glycosylation. *J Bacteriol* **189**, 8088-8098, doi:10.1128/JB.01318-07 (2007).
[5]Hug, I. *et al.* Exploiting bacterial glycosylation machineries for the synthesis of a Lewis antigen-containing glycoprotein. *J Biol Chem* **286**, 37887-37894, doi:10.1074/jbc.M111.287755 (2011).
[6]Price, N. L. *et al.* Glycoengineered Outer Membrane Vesicles: A Novel Platform for Bacterial Vaccines. *Sci Rep* **6**, 24931, doi:10.1038/srep24931 (2016).
[7]Kay, E. J., Yates, L. E., Terra, V. S., Cuccui, J. & Wren, B. W. Recombinant expression of *Streptococcus pneumoniae* capsular polysaccharides in *Escherichia coli*. *Open Biol* **6**, 150243, doi:10.1098/rsob.150243 (2016).

Supplementary Table 2

Primer	Sequence
igr F	5’ACTGGTCGACTAGTAGTACTATATGGCTTTAAA
igr R	5’ACTGCTGCAGTTAATATTCTATTGAACAAAATTTTAAC
H325A F	5’GAGAATGGTTTACATACTCAGCGAATTTGTTCTTAGATTTAATG
H325A R	5’CATTAAATCTAAGAACAAATTCGCTGAGTATGTAAACCATTCTC
S82A F – ADP1	5’GGAGTCCAAGAAATTGCGGCAAGTAATGCCA
S82A R– ADP1	5’GTGGCATTACTTGCCGCAATTTCTTGGACTCC
S84A F– ADP1	5’CAAGAAATTTCAGCAGCGAATGCCACTACGAAC
S84A R– ADP1	5’GTTCGTAGTGGCATTCGCTGCTGAAATTTCTTG
S82A 110254 F	5’ACAGATCGCGTCCGGCGCCgCAGCAGCGACAACAAATGTAGCGT
S82A 110254 R	5’ACGCTACATTTGTTGTCGCTGCTGcGGCGCCGGACGCGATCTGT
S79A 110254 F	5’CGGGCGTCACACAGATCGCGgCCGGCGCCTCAGCAGCGACAACA
S79A 110254 R	5’TGTTGTCGCTGCTGAGGCGCCGGcCGCGATCTGTGTGACGCCCG
