# Supplementary material for: A platform for glycoengineering a polyvalent pneumococcal bioconjugate vaccine using E. coli as a host
Source: Nat Commun. 2019 Feb 21;10:891. doi: 10.1038/s41467-019-08869-9 (PMC6385209; doi:10.1038/s41467-019-08869-9)
Supplement: Supplementary file 2 — Reporting Summary [file 41467_2019_8869_MOESM2_ESM.pdf]

nature research

Corresponding author(s): Mario Feldman

Last updated by author(s): Jan 28, 2019

Reporting Summary

Nature Research wishes to improve the reproducibility of the work that we publish. This form provides structure for consistency and transparency in reporting. For further information on Nature Research policies, see Authors & Referees and the Editorial Policy Checklist.

Statistics

For all statistical analyses, confirm that the following items are present in the figure legend, table legend, main text, or Methods section.

n/a	Confirmed	
☐	☒	The exact sample size (*n*) for each experimental group/condition, given as a discrete number and unit of measurement
☐	☒	A statement on whether measurements were taken from distinct samples or whether the same sample was measured repeatedly
☐	☒	The statistical test(s) used AND whether they are one- or two-sided<br>*Only common tests should be described solely by name; describe more complex techniques in the Methods section.*
☐	☒	A description of all covariates tested
☐	☒	A description of any assumptions or corrections, such as tests of normality and adjustment for multiple comparisons
☐	☒	A full description of the statistical parameters including central tendency (e.g. means) or other basic estimates (e.g. regression coefficient) AND variation (e.g. standard deviation) or associated estimates of uncertainty (e.g. confidence intervals)
☒	☐	For null hypothesis testing, the test statistic (e.g. *F*, *t*, *r*) with confidence intervals, effect sizes, degrees of freedom and *P* value noted<br>*Give P values as exact values whenever suitable.*
☒	☐	For Bayesian analysis, information on the choice of priors and Markov chain Monte Carlo settings
☒	☐	For hierarchical and complex designs, identification of the appropriate level for tests and full reporting of outcomes
☒	☐	Estimates of effect sizes (e.g. Cohen's *d*, Pearson's *r*), indicating how they were calculated

Our web collection on statistics for biologists contains articles on many of the points above.

Software and code

Policy information about availability of computer code

Data collection	GraphPad Prism 7 was used to perform statistical analyses
Data analysis	GraphPad Prism 7 was used to perform statistical analyses

For manuscripts utilizing custom algorithms or software that are central to the research but not yet described in published literature, software must be made available to editors/reviewers. We strongly encourage code deposition in a community repository (e.g. GitHub). See the Nature Research guidelines for submitting code & software for further information.

Data

Policy information about availability of data

All manuscripts must include a data availability statement. This statement should provide the following information, where applicable:

- Accession codes, unique identifiers, or web links for publicly available datasets
- A list of figures that have associated raw data
- A description of any restrictions on data availability

The authors declare that data supporting the findings of this study are available within the paper and its supplemental files. The source data underlying Figures 1, 3, 4, 5, 6, 7 and supplemental figures 2, and 5 are provided as a Source Data file.

Field-specific reporting

Please select the one below that is the best fit for your research. If you are not sure, read the appropriate sections before making your selection.

☒ Life sciences ☐ Behavioural & social sciences ☐ Ecological, evolutionary & environmental sciences

Life sciences study design

All studies must disclose on these points even when the disclosure is negative.

Sample size	All murine immunization experiments contained 10 mice per group with the exception of the third immunization experiment comparing the effects of a trivalent ComP-CPS8/9V/14 vaccine formulated in Alum (these groups contained 6 mice per group - three male and three female). This is based on past experience vaccinating mice with conjugate vaccines and on our sample size calculation as follows: using a two-sided unpaired Student's t test to compare two groups of mice with an alpha probability of 0.05, a power of 0.8, and a standard deviation of 0.131, a sample size of 3 mice per group is required to detect a statistically meaningful difference (calculations performed at https://www.stat.ubc.ca/~rollin/stats/ssize/n2.html) .
Data exclusions	For data associated with Supplemental Figure 5 (see Source Data as well) the Rout method for identifying outliers was performed and used to remove two data points from the IgG Placebo Day 49 bleed group as well as two points from the IgG CPS14-ComP Pre-bleed group.
Replication	All vaccines used in this study were prepared and purified multiple times and analyzed by western blot and Coomassie staining. Sera samples analyzed by ELISA were performed as technical triplicates to control for samples. All attempts at replication were successful.
Randomization	Mice groups were not randomized because clonal BALB/c mice were used in all vaccination groups.
Blinding	Investigators were blinded to vaccine groups; specifically, vaccines were arbitrarily numbered and were not disclosed to team members performing the vaccination until after the trials had been completed.

Reporting for specific materials, systems and methods

We require information from authors about some types of materials, experimental systems and methods used in many studies. Here, indicate whether each material, system or method listed is relevant to your study. If you are not sure if a list item applies to your research, read the appropriate section before selecting a response.

Materials & experimental systems

n/a	Involved in the study	
☐	☒	Antibodies
☒	☐	Eukaryotic cell lines
☒	☐	Palaeontology
☐	☒	Animals and other organisms
☒	☐	Human research participants
☒	☐	Clinical data

Methods

n/a	Involved in the study	
☒	☐	ChIP-seq
☒	☐	Flow cytometry
☒	☐	MRI-based neuroimaging

Antibodies

Antibodies used	Primary antibodies included: Pneumococcus Type 8 Serum (Ref. # 16751), Pneumococcus Type 9 Serum (Ref. # 16903), and Pneumococcus Type 14 Serum (Ref. # 16751) all used at 1:1000 dilutions. Additional antibodies included 6x-His Tag Monoclonal Antibody (HIS.H8) (Catalog # MA1-21315) used at 1:1000 and Anti-Pseudomonas Exotoxin A antibody (P2318-1ML) used at 1:5000. Secondary antibodies included Licor IRDye 680RD goat anti-mouse (925-68070) and goat anti-rabbit 800CW (926-32211) used at 1:10000 dilutions<br>ELISA Secondary antibody: HRP-linked IgG (Cell Signaling Technology # 7076) diluted 1:4000
Validation	Antibody validation can be found from each of the manufacturer's website by searching for the specific catalog number.

Animals and other organisms

Policy information about studies involving animals; ARRIVE guidelines recommended for reporting animal research

Laboratory animals	4-6 week old BALB/c mice were used for all studies involving vaccination experiments. Female mice were used for the monovalent ComP-CPS14 immunization described for Supplemental Figure 5A-C as well as for the Trivalent ComP-CPS8/9V/14 immunization described in Figure 4; Both male and female mice were used for a repeat of the ComP-CPS8/9V/14 immunization using Alum instead of Freund's adjuvant described in Figure 5 as well as immunizations for EPA-8 vaccinated mice described in Figure 7
Wild animals	The study did not involve wild animals
Field-collected samples	The study did not involve samples collected from the field
Ethics oversight	All murine immunizations complied with all relevant ethical regulations for animal testing and research. Immunizations were

Ethics oversight	conducted at the Southern Alberta Cancer Research Institute (SACRI) antibody services and Washington University School of Medicine in St. Louis according to institutional guidelines and received approval from the University of Calgary Animal Research and Education Executive Committee and the Institutional Animal Care and Use Committee at Washington University in St. Louis, respectively.

Note that full information on the approval of the study protocol must also be provided in the manuscript.
